# *Cells* Best Paper Award 2015

**DOI:** 10.3390/cells4030275

**Published:** 2015-07-15

**Authors:** Alexander E. Kalyuzhny

**Affiliations:** Editor-in-Chief, Neuroscience, UMN Twin Cities, 6-145 Jackson Hall, 321 Church St SE, Minneapolis, MN 55455, USA; E-Mail: kalyu001@umn.edu

*Cells* has instituted a “Best Paper” award to recognize the most outstanding papers in the area of cell biology, molecular biology, and biophysics, published in *Cells*. We are pleased to announce the first “*Cells* Best Paper Award” for 2015. The winners were chosen by the Editor-in-Chief and selected Editorial Board Members from all the research papers published in 2013 and 2014. We are pleased to announce that the following three papers have won the *Cells* Best Paper Award in 2015:

*First Prize*
  Tetyana Shandala, Chiaoxin Lim, Alexandra Sorvina and Douglas A. Brooks
    A Drosophila Model to Image Phagosome Maturation
    *Cells* **2013**, *2*(2), 188-201; doi:10.3390/cells2020188.
    Available online: http://www.mdpi.com/2073-4409/2/2/188/htm
    “There is a lot of research aimed at unraveling mechanism of phagosomes’ formation. Using Drosophila as a model is a very interesting approach.”—Dr. Alexander E. Kalyuzhny
    “Nice in vivo study showing Rab7 involvement in phagosome maturation.”—Dr. Heike Fölsch
*Second Prize*
  Sreedhar Thirumala, Jeffrey M. Gimble and Ram V. Devireddy
    Methylcellulose Based Thermally Reversible Hydrogel System for Tissue Engineering Applications
    *Cells* **2013**, *2*(3), 460-475; doi:10.3390/cells2030460
    Available online: http://www.mdpi.com/2073-4409/2/3/460/htm
    “Hydrogels are of interest to many applications in tissue engineering including stem cells.”—Dr. Alexander E. Kalyuzhny
*Third Prize*
  Isaac A. Rodriguez, Scott A. Sell, Jennifer M. McCool, Gunjan Saxena, Andrew J. Spence and Gary L. Bowlin
    A Preliminary Evaluation of Lyophilized Gelatin Sponges, Enhanced with Platelet-Rich Plasma, Hydroxyapatite and Chitin Whiskers for Bone Regeneration
    *Cells* **2013**, *2*(2), 244-265; doi:10.3390/cells2020244
    Available online: http://www.mdpi.com/2073-4409/2/2/244/htm
    “The paper dealing with tissue engineering/regeneration. Nice experimental design.”—Dr. Alexander E. Kalyuzhny

**Prize Awarding Committee**
  Editor-in-Chief
    Dr. Alexander E. Kalyuzhny
    Neuroscience, UMN Twin Cities, 6-145 Jackson Hall, 321 Church St SE, Minneapolis, MN 55455, USA
    E-Mail: kalyu001@umn.edu
  Editorial Board Member
    Dr. Heike Fölsch
    Department of Cell and Molecular Biology, Northwestern University, Feinberg School of Medicine, Ward 11-270, 303 East Chicago Avenue, Chicago, IL 60611, USA
    Email: h-folsch@northwestern.edu
  Editorial Board Member
    Dr. Vladimir V. Didenko
    Departments of Neurosurgery and Molecular and Cellular Biology, Baylor College of Medicine, Houston, TX 77030, USA
    Email: vdidenko@bcm.edu
  Editorial Board Member
    Prof. Dr. Jeremy C. Simpson
    University College Dublin, School of Biology and Environmental Science, Science Center - West, Belfield, Dublin 4, Ireland
    Email: jeremy.simpson@ucd.ie

We believe that these three exceptional papers are valuable contributions to the cell biology and molecular biology research field. On behalf of the Prize Awarding Committee and the Editorial Board of *Cells*, we would like to congratulate these teams for their excellent work. In recognition of their accomplishments, Dr. Tetyana Shandala, Dr. Ram V. Devireddy, and Dr. Gary L. Bowlin will receive the privilege of publishing a paper, free of charge, in Open Access format in *Cells*, respectively, after the usual peer-review procedure.

